# Is Laminectomy Desirable in Traumatic Compression of the Cord?

**Published:** 1892-05-28

**Authors:** 


### The Practitioner's Mirror and Retrospect.

[*The Editor will be glad to receive offers of co-operation and contributions from members of the profession. All letters should be addressed to* The Editor, The Lodge, Porchester Square, London, W.]

### MEDICINE.

### IS LAMINECTOMY DESIRABLE IN TRAUMATIC COMPRESSION OF THE CORD?

In fractures in any region of the spine with injury to the cord, the prognosis is so bad that treatment which will give any reasonable hope of relief ought to be welcome. Either in the cervical region, below the third vertebra, or in the upper dorsal region with paralysis of the intercostals and abdominal muscles, death from respiratory failure is very rapid, and even in the lower dorsal region, although the patient may live many months, bed sores form, and he will very likely die from surgical kidneys secondary to his cystitis.

Attempts at reduction of the displacement by extension and manipulation are very risky. They may cause greater injury to the cord than the original injury, and may thus, in the cervical region at any rate, quickly hasten the fatal result. In the dorsal region the spine is more fixed, so that even if the risk were less, we have less chance of effecting reduction in this way. In those rare cases of pure dislocation, the attempt at reduction is considered more justifiable.

As extension and manipulation has very little to recommend them in cases of fracture, the way is open to laminectony to establish a better claim to acceptance if it can. It is generally admitted that the risk of laminectomy if done antiseptically is not great. But here we are dealing with an almost hopeless condition, and even if the risks were greater they would not be great compared with the mortality of the untreated injury, which, taking all regions, has been shown to be 80 per cent. The question then is, What good can we hope to do by laminectomy? To this question replies are various. The American surgeons strongly advise it. Thorburn does not consider it offers any prospect of relief, either from *a priori* reasoning or from the result in published cases. Horsley says that the chance of its doing good is very small, but that seeing how hopeless the condition is we are right in giving that chance. Abbe thinks that although we cannot be hopeful about its doing much good, yet "we ought not to occupy the ultra conservative ground of the past in this field."

It is probable that one of the chief reasons why we cannot do more good by removing the arches against which the cord is compressed, is that it is not the compression which remains after the accident which chiefly damages the cord, but a much greater compression at the time of the accident, the displaced bones quickly returning, to some extent, into their normal position again. It is practically a sudden nipping of the cord, with permanent compression to a less extent, and the irritation of the rough bone sets up myelitis. We may, by laminectomy, relieve this after compression, and the risks of myelitis from irritation, but we cannot relieve the severe crushing which took place at the moment of the injury. Again, we have seen the cord much softened and ploughed up by hæmorrhage, and yet very little compressed, where there was also displacement of the vertebræ; and in such cases we should do no good. We could not distinguish the symptoms of such lesion from compression from the displaced vertebræ, for we could hardly say that the absence of signs of irritation of the nerve roots enable us to exclude paralysis from bony compression. In any case, then, in which we undertake laminectomy we may find the cord damaged in a way laminectomy cannot relieve. But it is the same in cerebral compression. How often is compression and laceration of the brain combined, and yet we do not hesitate to raise depressed bone in the presence of symptoms because we cannot tell how far the brain is also lacerated. We do what we can, fearing that it may turn out very little, and yet it may do much good. And so with injuries of the spine, even the "off chance" is worth having in so desperate a condition.

But with injuries of the corda equina, i.e., injuries below the first lumbar vertebra, we can do much more by laminectomy. Thorburn points out that here we have to deal practically with injuries of peripheral nerves, and the desirability of operating to remove compression seems generally admitted, but opinions differ as to whether it is wise to wait a few weeks to see if recovery takes place or to operate at once. Thorburn is in favour of waiting six weeks.

We have been writing as if all fractures of the spine with displacement were fractures through the bodies, because this is almost always so, but in a few rare cases of fracture with compression of the cord the bodies are not fractured, but only the laminæ. These are essentially cases for laminectomy. A case under the care of M. Péan in Paris very well illustrates this,* and it also shows how very latent the symptoms may be. A man was bitten by a horse in the middle of his back. He did not think much of it, and no symptoms came on for three days, and then paralysis with retention of urine and severe pain in the back set in. An irregularity in the mid-dorsal region of the spine was detected, and M. Péan found on operating the laminæ driven into the canal, and he removed ten fragments of bone. The patient was restored to almost his ordinary condition.

In his address on "The Surgery of the Central Nervous System," at the International Medical Congress of 1890, Horsley records six cases of laminectomy for fracture of the spine, but they were not operated on directly after the accident, the earliest period being five months after, so that they are not any guide to us in determining the value of early operative interference. Five out of the six were cases of complete paraplegia. The results were not very encouraging, as only sensation returned, not motion. No case, however, seems to have died of the operation.

Macewen's case of laminectomy for fracture, recorded in his address on "The Surgery of the Brain and Spinal Cord" at the British Medical Meeting in 1888, is a very interesting one, but here the operation was not done for several weeks after the accident. The back was injured at the level of the lower dorsal vertebræ, and absolute motor paralysis with incontinence resulted. Hyperæsthesia and great muscular atrophy set in, bed sores formed, and his urine became ammoniacal, and his temperature ran up. The arch of the twelfth dorsal vertebra was found slightly depressed, and extending from the eleventh dorsal to the second lumber on the posterior aspect of the dura mater was a quarter of an inch of thickened connective tissue. This Macewen cut away. The limbs became warmer the same night, and he began to move his toes on the third day. "He was soon able to walk with support, which a year subsequently he discarded, and now (1885) he can move about with ease, but with a paraplegic gait."

We have not so far referred to intraspinal traumatic hæmorrhage where the blood is outside the cord, and we are afraid it is of little use to do so. In the first place, how can a hæmorrhage outside the cord be so localised in the canal as to compress any one segment of the cord, when the communication up and down the canal is so free both within and without the dura mater; and in the second place, even if it could be localised, how can it be diagnosed from other causes

* *B. Med. Journal*, 1889, Vol. I., p. 672.

of so-called concussion symptoms, such as stretching of the cord? We do not write now of hæmorrhage within the cord; here, of course, we can do no good by laminectomy.

We cannot conclude this paper without a brief reference to Macewen's case* of injury to the cord—he thinks stretching and hæmorrhage into it—in which both arms were partially paralysed and all the intercostals completely paralysed, but not the legs, and Macewen throws out some suggestion with regard to the functions of the direct pyramidal tracts founded on this remarkable distribution of the paralysis in this and some similar cases. For more detailed information reference must be made to his report of the case.

It used to be stated (and is still) that the abolition of the reflexes in transverse lesions above the lumbar enlargement is due to shock to the cord in that region or secondary myelitis; but Bastian† has shown that clinical and pathological observation in these cases do not give us the same results as physiological experiments on animals—that the reflexes remain abolished. Mr. Bowlby, at the Med. Chirurg. Society, May 1890, contended that, "As in all cases of complete crushing of the spinal cord, the deep reflexes were lost, and as spinal cords so injured were incapable of repair, operative interference was only likely to be of use when the deep reflexes were not abolished, and when the spinal cord was therefore not completely severed."

